# The number of household members as a risk factor for peptic ulcer disease

**DOI:** 10.1038/s41598-021-84892-5

**Published:** 2021-03-05

**Authors:** Mi Hong Yim, Keun Ho Kim, Bum Ju Lee

**Affiliations:** Future Medicine Division, Korea Institute of Oriental Medicine, 1672 Yuseong-daero, Yuseong-gu, Daejeon, 34054 Republic of Korea

**Keywords:** Diseases, Health care, Medical research, Risk factors

## Abstract

Peptic ulcer disease (PUD) is caused by many sociodemographic and economic risk factors other than *H. pylori* infection. However, no studies reported an association between PUD and the number of household members. We showed the number of family members affected by PUD based on sex in a Korean population. This cross-sectional study used 1998–2009 data from the Korea National Health and Nutrition Examination Survey of the Korea Centers for Disease Control and Prevention. Multiple binary logistic regression models adjusted for confounders were constructed to analyze the association of PUD with the number of household members. The number of household members was associated with PUD, age, body mass index (BMI), waist circumference, systolic blood pressure, hemoglobin, glucose, location (urban/rural), income, education level, stress, current drinking, and smoking in both sexes. Men with other household members had a higher PUD risk compared to men or women living alone (reference), and the opposite was observed for women. Men with 4 household members had a higher PUD risk than men living alone in the model adjusted for age, BMI, income, location, education, and stress (OR = 2.04 [95% CI 1.28–3.27], *p* value = .003). Women with more than 6 household members had a lower PUD risk than women living alone in the adjusted model (OR = 0.50 [0.33–0.75], *p* value = .001). Women with more household members had a lower PUD risk. However, more men had PUD than women regardless of the number of household members.

## Introduction

Peptic ulcer disease (PUD) is a common digestive disorder that generally refers to an acid peptic injury in the stomach, duodenum, Meckel’s diverticulum, or esophagus^1,2^. Most studies on PUD focused on *Helicobacter pylori* (*H. pylori*) infection, which affects gastrointestinal diseases, such as PUD and gastritis^3–5^. However, people who are not infected with *H. pylori* have PUD, and many people infected with *H. pylori* do not develop PUD^1,6–10^. Peptic ulcers are related to various risk factors other than *H. pylori* infection, including socioeconomic, environmental, and psychological characteristics and other potential factors.

Numerous studies of sociodemographic characteristics and peptic ulcers identified various risk factors, such as age, low education, low socioeconomic status or low salary, household member crowding, unemployment, marital strain, a blue-collar household, meal intake regularity, breakfast skipping, smoking, heavy alcohol intake, high body mass index (BMI), nonsteroidal anti-inflammatory drugs (NSAIDs), musculoskeletal pain, headache, psychological and physical stress, and previous peptic ulcers^6,11–29^. For example, an important risk factor for PUD is cigarette smoking^10,13,14,23,25–29^. Smoking is a risk factor for chronic active ulcers or asymptomatic PUD in the United States^13^, Israel^14^, Taiwan^23^, Denmark^26^, and Norway^28^ and in American men of Japanese ancestry^27^. Alcohol intake was also associated with PUD^30–34^. However, several studies disagreed with the association of PUD with alcohol intake and smoking^22,27,29,35–37^. Low education level is a risk factor for PUD because education level was related to living conditions, such as lifestyle, diet, and social stress, and these conditions are part of the multifactorial etiology of PUD^22,23^. Similar to education level, low socioeconomic class or status is associated with PUD^14,22,27,38,39^. Populations in low socioeconomic class or status are linked to heavy alcohol intake, smoking, hard physical work, hygiene, concerns about dismissal, inadequate nutrition, use of painkillers, and psychological stress^38,39^. Psychological stress and physical stress affect the development of ulcers because stress aggravates gastroduodenal blood flow, reduces acid buffering in the duodenum, and diminishes gastric hypersecretion^6,16^. Stress tends to be uncontrolled and unpredictable^38,40^, promotes the onset of disease^6,41^, and is one of the most common risk factors for PUD^28^.

A large number of studies revealed various risk factors for PUD, but no studies revealed an association between the number of household members and PUD or sex difference in this association. We hypothesized that women would be more likely to have PUD than men as the number of household members increased because women are more involved and exposed to more stressors in family affairs. Several risk factors, such as cigarette smoking, alcohol intake, obesity, and age, remain controversial. The present study focused on the association of PUD with the number of household members among various socioeconomic risk factors other than H. pylori infection in a Korean population. Notably, the findings revealed strong positive and negative associations between PUD and the number of household members according to sex.

## Results

### Characteristics of the subjects across categories of the number of household members

Table 1 indicates the sex differences between men and women and general characteristics of the PUD and non-PUD groups. Participant characteristics according to categories of the number of household members are indicated in Table 2 for men and Table 3 for women. A significant relationship between PUD and the number of household members was revealed for men. Age, SBP, location, education level, stress, current drinking, and smoking were also significantly associated with the number of household members. Men with 2 household members (7.14%) were more likely to have PUD and high SBP, to be less educated, and older than men in other household sizes. For women, PUD, age, SBP, location, education level, stress, current drinking and smoking had a statistically significant relationship to the number of household members. Women living alone (8.73%) were more likely to have PUD and high SBP, to be less educated, and older than women in other household sizes.

**Table 1:** General characteristics of the subjects in this study.

Variables	Men		*p* value	Women		*p* value
	Non-PUD	PUD		Non-PUD	PUD	
Number of subjects	13,293	904		17,747	1,078	
Age (years)^†^	41.49 ± 0.18	49.05 ± 0.54	< .001	43.36 ± 0.19	51.07 ± 0.57	< .001
BMI (kg/m^2^)^†^	23.81 ± 0.04	23.36 ± 0.12	< .001	23.17 ± 0.04	23.26 ± 0.12	.504
Waist circumference (cm)^†^	83.55 ± 0.11	83.62 ± 0.34	.846	77.83 ± 0.12	79.17 ± 0.36	< .001
SBP (mmHg)^†^	122.08 ± 0.20	122.73 ± 0.69	.351	116.31 ± 0.21	120.33 ± 0.71	< .001
DBP (mmHg)^†^	80.02 ± 0.14	79.48 ± 0.45	.237	74.27 ± 0.13	75.36 ± 0.39	.005
Pulse rate (beats per minute)^†^	17.46 ± 0.03	17.38 ± 0.09	.414	17.85 ± 0.03	17.83 ± 0.09	.781
Hemoglobin (mg/dl)^†^	15.22 ± 0.01	15.11 ± 0.05	.031	12.85 ± 0.01	12.87 ± 0.04	.659
Cholesterol (mg/dl)	185.13 ± 0.40	186.68 ± 1.42	.290	185.22 ± 0.35	189.94 ± 1.27	< .001
Triglycerides (mg/dl)^†^	150.59 ± 1.19	155.05 ± 4.61	.339	112.00 ± 0.73	118.15 ± 2.78	.029
Glucose (mg/dl)^†^	97.41 ± 0.24	99.53 ± 1.01	.039	95.18 ± 0.22	97.04 ± 0.83	.024
Creatinine (mg/dl)^†^	1.03 ± 0.002	1.01 ± 0.01	.006	0.82 ± 0.002	0.82 ± 0.01	.247
**Number of household members**^†^			< .001			< .001
1 member	5.18 (0.34)	2.71 (0.61)		6.28 (0.26)	11.91 (1.08)	
2 members	16.98 (0.40)	22.96 (1.61)		17.21 (0.39)	25.00 (1.51)	
3 members	25.05 (0.51)	24.11 (1.73)		23.26 (0.43)	21.87 (1.45)	
4 members	35.03 (0.60)	35.57 (1.99)		33.37 (0.55)	26.16 (1.61)	
5 members	12.02 (0.39)	9.72 (1.11)		13.33 (0.39)	10.68 (1.19)	
> = 6 members	5.73 (0.33)	4.94 (0.83)		6.55 (0.34)	4.37 (0.76)	
**Region (city)**			< .001			< .001
Seoul	21.79 (0.71)	14.66 (1.58)		22.08 (0.66)	16.79 (1.74)	
Busan	7.62 (0.48)	8.10 (1.12)		7.84 (0.49)	9.21 (1.33)	
Daegu	5.20 (0.42)	6.30 (1.12)		5.39 (0.41)	5.50 (0.97)	
Incheon	5.35 (0.41)	4.55 (0.84)		5.58 (0.39)	4.16 (0.94)	
Gwangju	3.19 (0.38)	3.46 (0.76)		3.01 (0.32)	3.49 (0.90)	
Daejeon	2.62 (0.31)	3.07 (0.71)		2.84 (0.29)	2.99 (0.87)	
Ulsan	1.77 (0.32)	2.67 (1.13)		1.78 (0.34)	1.48 (0.38)	
Gyeonggi-do	21.59 (0.67)	21.36 (1.95)		21.13 (0.63)	17.54 (1.76)	
Gangwon-do	3.54 (0.36)	1.34 (0.49)		3.23 (0.33)	1.65 (0.41)	
Chungcheongbuk-do	3.25 (0.40)	3.07 (0.75)		3.10 (0.38)	5.67 (1.34)	
Chungcheongnam-do	3.62 (0.44)	5.12 (0.96)		3.54 (0.41)	6.09 (1.23)	
Jeollabuk-do	3.31 (0.42)	4.26 (0.91)		3.32 (0.38)	4.31 (0.81)	
Jeollanam-do	3.59 (0.42)	4.68 (0.95)		3.67 (0.42)	4.70 (0.85)	
Gyeongsangbuk-do	5.66 (0.54)	8.16 (1.26)		5.62 (0.51)	7.46 (1.29)	
Gyeongsangnam-do	6.56 (0.62)	7.47 (1.21)		6.58 (0.59)	6.84 (1.05)	
Jeju-do	1.33 (0.25)	1.71 (0.60)		1.29 (0.22)	2.11 (0.55)	
**Location**			.009			< .001
Dong (urban)	80.89 (0.86)	76.82 (1.80)		81.14 (0.78)	73.34 (1.76)	
Eup, Myeon (rural)	19.11 (0.86)	23.18 (1.80)		18.86 (0.78)	26.66 (1.76)	
**Income**			.354			0.483
1st quartile (low)	23.57 (0.55)	24.56 (1.68)		23.32 (0.52)	25.11 (1.66)	
2nd quartile (lower-middle)	25.00 (0.49)	25.97 (1.74)		25.15 (0.45)	25.07 (1.51)	
3rd quartile (upper-middle)	25.73 (0.49)	22.57 (1.60)		25.49 (0.43)	23.31 (1.49)	
4th quartile (high)	25.71 (0.64)	26.89 (1.82)		26.04 (0.62)	26.51 (1.71)	
**Education**^†^			< .001			< .001
Elementary school or less	11.88 (0.34)	21.65 (1.55)		25.11 (0.50)	44.63 (1.87)	
Middle school	10.10 (0.31)	13.74 (1.22)		11.02 (0.28)	13.12 (1.29)	
High school	42.38 (0.61)	34.28 (1.92)		38.87 (0.51)	27.38 (1.66)	
University or higher	35.64 (0.63)	30.34 (1.94)		25.00 (0.52)	14.88 (1.54)	
Gastric cancer	0.27 (0.04)	0.71 (0.31)	.035	0.25 (0.04)	0.27 (0.14)	.876
Liver cancer^†^	0.09 (0.03)	0 (0)	.437	0.01 (0.01)	0.06 (0.06)	.032
Colorectal cancer*	0.17 (0.04)	0.54 (0.33)	.054	0.07 (0.03)	0.04 (0.04)	.494
Diabetes	4.55 (0.19)	7.02 (1.03)	.005	4.30 (0.17)	5.06 (0.70)	.250
Hypertension**	10.87 (0.31)	15.35 (1.35)	< .001	12.22 (0.31)	16.67 (1.31)	< .001
Stress**			.001			< .001
Extremely	5.42 (0.23)	7.35 (0.98)		5.82 (0.22)	9.64 (1.01)	
Very	25.40 (0.45)	30.96 (1.87)		26.96 (0.39)	35.76 (1.71)	
Slightly	54.22 (0.52)	48.59 (2.08)		52.77 (0.46)	43.64 (1.84)	
Rarely	14.97 (0.39)	13.09 (1.31)		14.45 (0.33)	10.96 (1.03)	
**Drinking, current**^†^			.001			< .001
Yes	86.48 (0.36)	82.09 (1.45)		65.08 (0.47)	55.23 (1.75)	
No	13.52 (0.36)	17.91 (1.45)		34.92 (0.47)	44.77 (1.75)	
**Smoking**^†^			< .001			.063
Current	53.13 (0.55)	55.39 (1.93)		6.07 (0.23)	8.27 (1.09)	
Former	26.69 (0.46)	31.31 (1.84)		4.77 (0.22)	4.86 (0.78)	
Never	20.19 (0.44)	13.3 (1.38)		89.16 (0.33)	86.88 (1.34)	

*BMI* body mass index, *SBP* systolic blood pressure, *DBP* diastolic blood pressure. *: *p* < 0.05; **: *p* < 0.01; ^†^*p* < 0.001. *, ** and † indicate *p* values for the sex difference. Continuous variables are summarized as the means ± SE (standard error). Categorical variables are summarized as percentages (SE). *p* values were obtained from a general linear model for continuous variables and from Rao-Scott chi-squared tests for categorical variables between the group without PUD and the group with PUD. All statistical analyses were performed using weight parameters, cluster parameters and stratification parameters to account for the complex sampling design.

**Table 2:** General characteristics of the subjects across categories of the number of household members for Korean men.

Variables	1 member	2 members	3 members	4 members	5 members	> = 6 members
Number of subjects	659	3,163	3,353	4,590	1,595	837
Peptic ulcer disease^†^	2.89 (0.63)	7.14 (0.53)	5.19 (0.42)	5.46 (0.38)	4.39 (0.52)	4.67 (0.77)
Age (years)^†^	38.40 ± 0.85	49.65 ± 0.47	41.29 ± 0.30	39.33 ± 0.18	40.62 ± 0.38	42.58 ± 0.60
BMI (kg/m^2^)**	23.35 ± 0.16	23.59 ± 0.07	23.89 ± 0.07	23.84 ± 0.05	23.84 ± 0.10	23.75 ± 0.17
Waist circumference (cm)^†^	81.48 ± 0.42	84.00 ± 0.21	83.75 ± 0.20	83.47 ± 0.15	83.49 ± 0.28	83.88 ± 0.45
SBP (mmHg)^†^	121.64 ± 0.68	125.51 ± 0.40	122.27 ± 0.36	120.28 ± 0.26	121.6 ± 0.47	123.97 ± 0.68
DBP (mmHg)	79.47 ± 0.52	79.73 ± 0.27	80.11 ± 0.24	80.11 ± 0.21	79.80 ± 0.33	80.39 ± 0.50
Pulse rate (beats per minute)*	17.59 ± 0.12	17.49 ± 0.06	17.44 ± 0.06	17.35 ± 0.05	17.55 ± 0.07	17.68 ± 0.12
Hemoglobin (mg/dl)^†^	15.24 ± 0.05	15.03 ± 0.03	15.24 ± 0.02	15.28 ± 0.02	15.22 ± 0.04	15.23 ± 0.05
Cholesterol (mg/dl)	182.01 ± 1.94	184.22 ± 0.74	185.30 ± 0.74	186.18 ± 0.61	184.62 ± 1.04	185.95 ± 1.52
Triglycerides (mg/dl)	139.24 ± 4.8	150.28 ± 2.62	149.59 ± 2.25	152.45 ± 1.98	151.36 ± 3.18	157.19 ± 5.49
Glucose (mg/dl)^†^	96.09 ± 1.02	100.14 ± 0.52	97.67 ± 0.45	96.18 ± 0.34	97.38 ± 0.65	98.69 ± 1.03
Creatinine (mg/dl)	1.03 ± 0.01	1.03 ± 0.01	1.03 ± 0.004	1.03 ± 0.005	1.02 ± 0.004	1.03 ± 0.01
**Location**^†^						
Dong (urban)	85.68 (1.69)	69.55 (1.35)	81.56 (1.11)	85.04 (1.07)	80.66 (1.52)	79.21 (2.07)
Eup, Myeon (rural)	14.32 (1.69)	30.45 (1.35)	18.44 (1.11)	14.96 (1.07)	19.34 (1.52)	20.79 (2.07)
**Income**^†^						
1st quartile (low)	31.27 (2.78)	28.09 (1.06)	21.46 (0.95)	20.40 (0.80)	27.22 (1.50)	25.09 (2.11)
2nd quartile (lower-middle)	15.52 (1.57)	24.36 (0.92)	26.75 (0.99)	25.11 (0.82)	26.94 (1.40)	23.77 (2.04)
3rd quartile (upper-middle)	22.47 (1.97)	22.02 (0.90)	25.67 (0.96)	28.3 (0.84)	23.3 (1.29)	26.41 (1.99)
4th quartile (high)	30.73 (2.67)	25.52 (1.08)	26.12 (1.03)	26.19 (0.99)	22.54 (1.45)	24.74 (2.30)
**Education**^†^						
Elementary school or less	13.04 (1.42)	26.44 (0.99)	12.30 (0.61)	6.10 (0.39)	10.47 (0.82)	12.51 (1.16)
Middle school	9.44 (1.15)	13.85 (0.70)	10.41 (0.61)	8.53 (0.43)	9.79 (0.83)	11.65 (1.31)
High school	46.31 (2.5)	34.18 (1.25)	41.47 (1.12)	45.47 (0.88)	42.58 (1.49)	40.79 (2.08)
University or higher	31.2 (2.63)	25.53 (1.12)	35.82 (1.09)	39.9 (0.93)	37.16 (1.54)	35.05 (2.12)
**Stress**^†^						
Extremely	6.15 (1.01)	5.66 (0.52)	4.53 (0.38)	5.81 (0.40)	6.04 (0.65)	6.10 (0.96)
Very	23.80 (1.98)	21.25 (0.92)	25.45 (0.90)	27.86 (0.77)	28.28 (1.39)	23.22 (1.74)
Slightly	52.63 (2.34)	49.87 (1.09)	55.65 (0.99)	55.15 (0.89)	52.90 (1.49)	54.19 (2.03)
Rarely	17.42 (1.67)	23.22 (0.97)	14.37 (0.72)	11.18 (0.56)	12.78 (0.93)	16.49 (1.57)
**Drinking, current**^†^						
Yes	87.09 (1.40)	79.97 (0.83)	85.84 (0.68)	89.33 (0.52)	87.34 (0.96)	84.96 (1.44)
No	12.91 (1.40)	20.03 (0.83)	14.16 (0.68)	10.67 (0.52)	12.66 (0.96)	15.04 (1.44)
**Smoking**^†^						
Current	56.34 (2.21)	49.49 (1.11)	52.17 (1.08)	54.35 (0.88)	55.63 (1.47)	54.91 (2.14)
Former	18.16 (1.59)	32.87 (1.04)	27.35 (0.87)	25.42 (0.72)	25.31 (1.26)	27.60 (2.05)
Never	25.50 (1.99)	17.64 (0.82)	20.48 (0.90)	20.23 (0.72)	19.06 (1.24)	17.49 (1.70)

*BMI* body mass index; *SBP* systolic blood pressure, *DBP* diastolic blood pressure. *: *p* < .05; **: *p* < .01; ^†^*p* < .001. *, ** and † indicate *p* values of the difference across categories of the number of household members. These *p* values were obtained from a general linear model for continuous variables and from Rao-Scott chi-squared tests for categorical variables. Continuous variables are summarized as the means ± SE (standard error). Categorical variables are summarized as percentages (SE). All statistical analyses were performed using weight, cluster and stratification parameters to account for the complex sampling design.

**Table 3:** General characteristics of the subjects across categories of the number of household members in Korean women.

Variables	1 member	2 members	3 members	4 members	5 members	> = 6 members
Number of subjects	1,580	3,883	4,127	5,738	2,333	1,164
Peptic ulcer disease^†^	8.73 (0.79)	6.83 (0.47)	4.53 (0.35)	3.81 (0.28)	3.89 (0.44)	3.26 (0.55)
Age (years)^†^	57.19 ± 0.99	51.53 ± 0.42	43.08 ± 0.29	38.63 ± 0.19	41.10 ± 0.37	42.55 ± 0.59
BMI (kg/m^2^)^†^	23.48 ± 0.12	23.71 ± 0.08	23.07 ± 0.06	22.90 ± 0.05	23.13 ± 0.08	23.31 ± 0.14
Waist circumference (cm)^†^	80.25 ± 0.42	80.48 ± 0.23	77.56 ± 0.18	76.36 ± 0.16	77.48 ± 0.26	78.37 ± 0.37
SBP (mmHg)^†^	126.86 ± 0.84	122.77 ± 0.44	115.65 ± 0.34	112.13 ± 0.27	115.09 ± 0.44	117.21 ± 0.73
DBP (mmHg)^†^	76.90 ± 0.41	76.67 ± 0.23	74.07 ± 0.21	72.92 ± 0.19	73.91 ± 0.28	74.21 ± 0.39
Pulse rate (beats per minute)	17.97 ± 0.08	17.76 ± 0.06	17.79 ± 0.05	17.86 ± 0.04	17.94 ± 0.06	17.98 ± 0.10
Hemoglobin (mg/dl)^†^	13.01 ± 0.03	12.98 ± 0.02	12.85 ± 0.02	12.80 ± 0.02	12.75 ± 0.03	12.79 ± 0.04
Cholesterol (mg/dl)^†^	195.58 ± 1.28	195.02 ± 0.83	185.81 ± 0.65	179.91 ± 0.57	181.87 ± 0.82	183.33 ± 1.23
Triglycerides (mg/dl)^†^	126.68 ± 2.57	129.42 ± 1.71	110.95 ± 1.27	102.51 ± 1.12	108.69 ± 1.75	113.26 ± 2.42
Glucose (mg/dl)^†^	97.96 ± 0.71	98.68 ± 0.48	94.09 ± 0.36	93.84 ± 0.35	94.51 ± 0.50	96.33 ± 0.72
Creatinine (mg/dl)^†^	0.84 ± 0.01	0.82 ± 0.004	0.81 ± 0.003	0.81 ± 0.003	0.82 ± 0.004	0.83 ± 0.01
**Location**^†^						
Dong (urban)	70.13 (1.72)	70.94 (1.26)	83.17 (0.95)	86.19 (0.93)	82.60 (1.33)	78.20 (2.14)
Eup, Myeon (rural)	29.87 (1.72)	29.06 (1.26)	16.83 (0.95)	13.81 (0.93)	17.40 (1.33)	21.80 (2.14)
**Income**^†^						
1st quartile (low)	40.47 (1.79)	27.01 (0.91)	22.07 (0.85)	18.47 (0.7)	24.37 (1.19)	24.36 (1.89)
2nd quartile (lower-middle)	28.83 (1.35)	25.9 (0.89)	25.36 (0.88)	24.33 (0.75)	25.69 (1.22)	21.64 (1.69)
3rd quartile (upper-middle)	18.63 (1.21)	22.35 (0.8)	25.79 (0.85)	29.51 (0.78)	21.92 (1.05)	25.09 (1.77)
4th quartile (high)	12.08 (1.13)	24.74 (0.91)	26.78 (0.99)	27.7 (0.9)	28.02 (1.44)	28.91 (2.2)
**Education**^†^						
Elementary school or less	61.87 (2.09)	46.61 (1.11)	23.74 (0.82)	11.68 (0.49)	20.46 (0.94)	26.88 (1.43)
Middle school	7.96 (0.81)	12.91 (0.64)	12.22 (0.56)	10.70 (0.47)	9.83 (0.65)	10.24 (1.05)
High school	17.95 (1.94)	23.89 (0.91)	38.22 (0.96)	47.77 (0.80)	43.48 (1.25)	39.76 (1.62)
University or highly	12.22 (1.20)	16.59 (0.94)	25.82 (0.85)	29.85 (0.82)	26.23 (1.25)	23.11 (1.58)
**Stress**^†^						
Extremely	6.99 (0.85)	7.04 (0.50)	5.99 (0.43)	5.15 (0.36)	6.77 (0.66)	4.94 (0.76)
Very	27.01 (1.36)	26.81 (0.81)	27.28 (0.74)	27.29 (0.69)	28.2 (1.07)	28.54 (1.49)
Slightly	39.94 (1.4)	46.94 (0.93)	52.99 (0.92)	56.75 (0.75)	53.21 (1.21)	52.80 (1.79)
Rarely	26.07 (1.37)	19.20 (0.72)	13.74 (0.64)	10.81 (0.51)	11.83 (0.74)	13.72 (1.28)
**Drinking, current**^†^						
Yes	51.79 (1.65)	56.24 (0.97)	64.36 (0.89)	71.87 (0.72)	65.07 (1.16)	63.25 (1.80)
No	48.21 (1.65)	43.76 (0.97)	35.64 (0.89)	28.13 (0.72)	34.93 (1.16)	36.75 (1.80)
**Smoking**^†^						
Current	15.19 (1.13)	8.66 (0.63)	6.02 (0.47)	4.03 (0.34)	4.57 (0.51)	5.06 (0.78)
Former	7.36 (0.80)	4.70 (0.43)	5.50 (0.43)	4.24 (0.37)	4.14 (0.47)	3.80 (0.70)
Never	77.45 (1.29)	86.64 (0.73)	88.48 (0.63)	91.73 (0.50)	91.3 (0.69)	91.14 (1.00)

*BMI* body mass index, *SBP* systolic blood pressure, *DBP* diastolic blood pressure. *: *p* < .05; **: *p* < .01; ^†^*p* < .001. *, ** and † indicate *p* values of the difference across categories of the number of household members. These *p* values were obtained from a general linear model for continuous variables and from Rao-Scott chi-squared tests for categorical variables. Continuous variables are summarized as the means ± SE (standard error). Categorical variables are summarized as percentages (SE). All statistical analyses were performed using weight, cluster and stratification parameters to account for the complex sampling design.

### Associations between PUD and the number of household members

Table 4 presents the association of PUD with the number of household members for model comparison with adjustment for covariates. The number of household members was significantly associated with PUD risk for men and women with and without adjustment. These models showed very different trends according to sex. Men with other household members had a higher risk of PUD in all models compared to men living alone (reference group). Specifically, men with 4 household members had a higher risk of PUD than men living alone in model 1 (adjusted for age and BMI) (OR = 2.09 (1.31–3.35), *p* value = 0.002), model 2 (adjusted for age, BMI, income, location, and education) (OR = 2.13 (1.33–3.40), *p* value = 0.002), and model 3 (for age, BMI, income, location, education, and stress) (OR = 2.04 (1.28–3.27), *p* value = 0.003). Notably, the risk of PUD decreased for women as the number of household members increased compared to women living alone in most models. Women with more than 6 household members had a lower risk of PUD than women living alone in the crude model (OR = 0.35 (0.24–0.52), *p* value < 0.001), model 1 (OR = 0.52 (0.35–0.77), *p* value = 0.001), model 2 (OR = 0.51 (0.34–0.76), *p* value = 0.001), and model 3 (OR = 0.50 (0.33–0.75), *p* value = 0.001).

**Table 4:** Adjusted odds ratios for PUD according to the number of household members.

Model	Variables	Men	*p* value	Women	*p* value
		OR (95% CI)		OR (95% CI)	
Crude	Number of household members		< .001		< .001
	1 member (Reference)	1		1	
	2 members	2.59 (1.63–4.10)	< .001	0.77 (0.61–0.97)	.027
	3 members	1.84 (1.15–2.96)	.011	0.50 (0.39–0.64)	< .001
	4 members	1.94 (1.21–3.11)	.006	0.41 (0.32–0.53)	< .001
	5 members	1.55 (0.93–2.57)	.092	0.42 (0.31–0.58)	< .001
	> = 6 members	1.65 (0.94–2.89)	.081	0.35 (0.24–0.52)	< .001
Model 1	Number of household members		.020		.004
	1 member (Reference)	1		1	
	2 members	1.80 (1.13–2.87)	.013	0.92 (0.72–1.17)	.487
	3 members	1.76 (1.10–2.83)	.018	0.73 (0.56–0.96)	.025
	4 members	2.09 (1.31–3.35)	.002	0.70 (0.53–0.92)	.012
	5 members	1.55 (0.94–2.58)	.089	0.66 (0.48–0.90)	.009
	> = 6 members	1.49 (0.85–2.62)	.162	0.52 (0.35–0.77)	.001
Model 2	Number of household members		.016		.009
	1 member (Reference)	1		1	
	2 members	1.79 (1.13–2.86)	.014	0.89 (0.70–1.14)	.362
	3 members	1.78 (1.11–2.85)	.017	0.74 (0.56–0.97)	.031
	4 members	2.13 (1.33–3.40)	.002	0.72 (0.55–0.95)	.022
	5 members	1.56 (0.94–2.59)	.086	0.66 (0.48–0.91)	.011
	> = 6 members	1.50 (0.86–2.63)	.156	0.51 (0.34–0.76)	.001
Model 3	Number of household members		.026		.004
	1 member (Reference)	1		1	
	2 members	1.81 (1.14–2.88)	.012	0.86 (0.67–1.10)	.235
	3 members	1.76 (1.10–2.81)	.018	0.71 (0.54–0.93)	.012
	4 members	2.04 (1.28–3.27)	.003	0.69 (0.53–0.91)	.009
	5 members	1.50 (0.90–2.48)	.117	0.63 (0.46–0.86)	.004
	> = 6 members	1.51 (0.86–2.64)	.152	0.50 (0.33–0.75)	.001

*OR* odds ratio, *CI* Confidence interval. Model 1: Adjusted for age and body mass index. Model 2: Adjusted for age, body mass index, income, location, and education. Model 3: Adjusted for age, body mass index, income, location, education, and stress. *p* values were obtained from multiple logistic regression analyses with adjustment. These analyses were performed using weight, cluster and stratification parameters to account for the complex sampling design. Odds ratios are presented with 95% confidence intervals.

## Discussion

Gastric and duodenal ulcer diseases have been studied for a long time worldwide. However, there were no previous studies on the number of household members and PUD. Therefore, we reviewed the literature on family affairs closely in relation to the number of household members, PUD, and sex differences, and we expected that women would have more PUD than men because women are more involved and experience more stress in family affairs than men^42^. The regularity of meal intake and skipping breakfast have a strong effect on PUD^11,15,16,25,43^, and the number of household members is closely associated with the regularity of meal intake, meal preparation, and the role of meal production due to the common activities among family members^11,25,43,44^. For example, Leblanc et al.^45^ examined sex differences in eating behaviors and dietary intake based on a food frequency questionnaire and the Three‐Factor Eating questionnaire, and they concluded that women engaged in meal preparation each week much more frequently than men. Ma^46^ and Quelly^47^ noted that women have more responsibility for meal preparation than men in many countries and cultures. Therefore, women play an important role in meal production, ingredient purchases, cooking methods, and decisions on the type, nutrition, and quantity of meals for adults and children in their family^14,19^. Many adult women in Korea secure a job to obtain income in addition to preparing most meals and handling family-related activities, such as house cleaning and washing. Therefore, women expend time and labor and are more stressed in the preparation of meals as the number of household members increases, but this finding is not universal. Despite some ongoing changes, Korea remains a patriarchal society. Therefore, we hypothesized that women would be more likely to have PUD than men as the number of household members increased. However, our results were the opposite of what we expected. Our findings indicated that women were less likely to have PUD as the number of household members increased, and men were more likely regardless of the number of family members. Further studies are needed to clarify this finding.

Several studies suggested that alcohol intake was a risk factor for PUD^30,33,34^ or the occurrence of PUD^31,32^, but other studies argued that alcohol intake was not associated with PUD^22,27,29,35–37^. Kato et al.^27^ reported that alcohol intake was not a risk factor for gastric or duodenal ulcers in Hawaii. Chou^36^ suggested that moderate alcohol intake minimally increased the odds of PUD in a large U.S. population study. Johnsen et al.^22^ argued that alcohol and coffee intake were not associated with PUD in a 7-year follow-up study in Norway. Levenstein^16^ argued that these controversial results may be due to the total amount of alcohol intake and indicated that moderate intake seemed to strengthen gastroduodenal mucosa, but heavy alcohol intake may cause PUD due to the direct mucosal and acid secretion stimulation. Liu et al.^48^ argued that moderate alcohol intake was related to a reduction of H. pylori infection. Our findings are consistent with the results of previous studies^30,33,34^ and indicated that alcohol drinking was highly associated with PUD in men and women in crude analyses and that this risk factor showed significant differences according to the number of household members.

Numerous studies reported that smoking was an important risk factor for PUD^10,13,14,22,23,25–29^. However, some researchers disagreed with the association between smoking and PUD^35,37^. Aldoori et al.^35^ demonstrated that current and past smoking was not associated with the risk of duodenal ulcers despite adjustment for age, BMI, dietary fiber, and the use of drugs, such as aspirin and nonsteroidal anti-inflammatory drugs, in a prospective study with 6 years of follow-up. Our findings differed slightly from previous studies depending on sex^10,13,14,22,23,25–29,35,37^. Our results showed that smoking was highly associated with PUD in men but not women.

The relationship between PUD and abdominal adiposity or obesity is not clear^19,20^. Several studies suggested that high BMI was an independent risk factor for PUD^20,23,24^, and other studies reported that BMI was not related to PUD^49,50^. Our results showed that BMI was associated with PUD in men but not women, and waist circumference was strongly related to PUD in women but not men.

Many previous studies suggested that age was one of the most reliable risk factors for PUD^10,13,14,17,19–22^. Although the incidence of PUD is decreasing in many countries due to new therapies^1^, the incidence of PUD and its bleeding complications is growing in the elderly population, and PUD mortality, management, and hospitalization are increasing due to the rapid population increase in most countries^1,21,51^. However, one study argued that age, sex, and abdominal symptoms were not risk factors for PUD in Japanese patients^37^. Our findings revealed that age was very strongly associated with PUD in men and women.

### Limitations of the study

This study had several limitations. First, we did not determine why men were at higher risk of PUD than women regardless of the number of household members or why women were at lower risk of PUD as the number of household members increased. It is difficult to find causality in the results due to the cross-sectional nature of this study. Further longitudinal studies are needed to identify causal relations. Second, our findings may not be similar to other countries or ethnic groups due to the differences in socioeconomic, environmental, and psychological characteristics. Last, many studies reported that H. pylori infection was highly associated with PUD. However, our study did not consider the effects of H. pylori infection because information of H. pylori infection was not provided in the Korea National Health and Nutrition Examination Survey. Therefore, a limitation of this study is that H. pylori infection was not considered. Further study is needed to reveal the causes of different associations between the number of household members and risk of PUD in men and women and to consider the effects of H. pylori infection. Despite these limitations, this study also has strengths. The statistical results in this study are powerful because the KNHANES provides a nationally representative sample of the Korean population. To our knowledge, this study is the first report of a significant association between PUD and the number of household members in the world.

## Methods

### Sampling and data source

This cross-sectional study used data from the Korea National Health and Nutrition Examination Survey (KNHANES I-IV) from 1998 to 2009, which included PUD diagnosis. The KNHANES has been performed by the Korea Centers for Disease Control and Prevention (KCDC) since 1998. The purpose of the KNHANES is to produce representative and reliable statistics of national and municipal units of the health, food and nutrition intake of the population and to develop health promotion programs. The present study selected the KNHANES sample using the multistage stratified cluster sampling method, which is a complex sampling design method, to improve the sample representativeness and estimation accuracy. For complex sample analysis, three elements of complex sample design must be considered: weight, cluster and stratification variables. These three variables are provided in the source database (http://knhanes.cdc.go.kr/).

**Figure 1:**
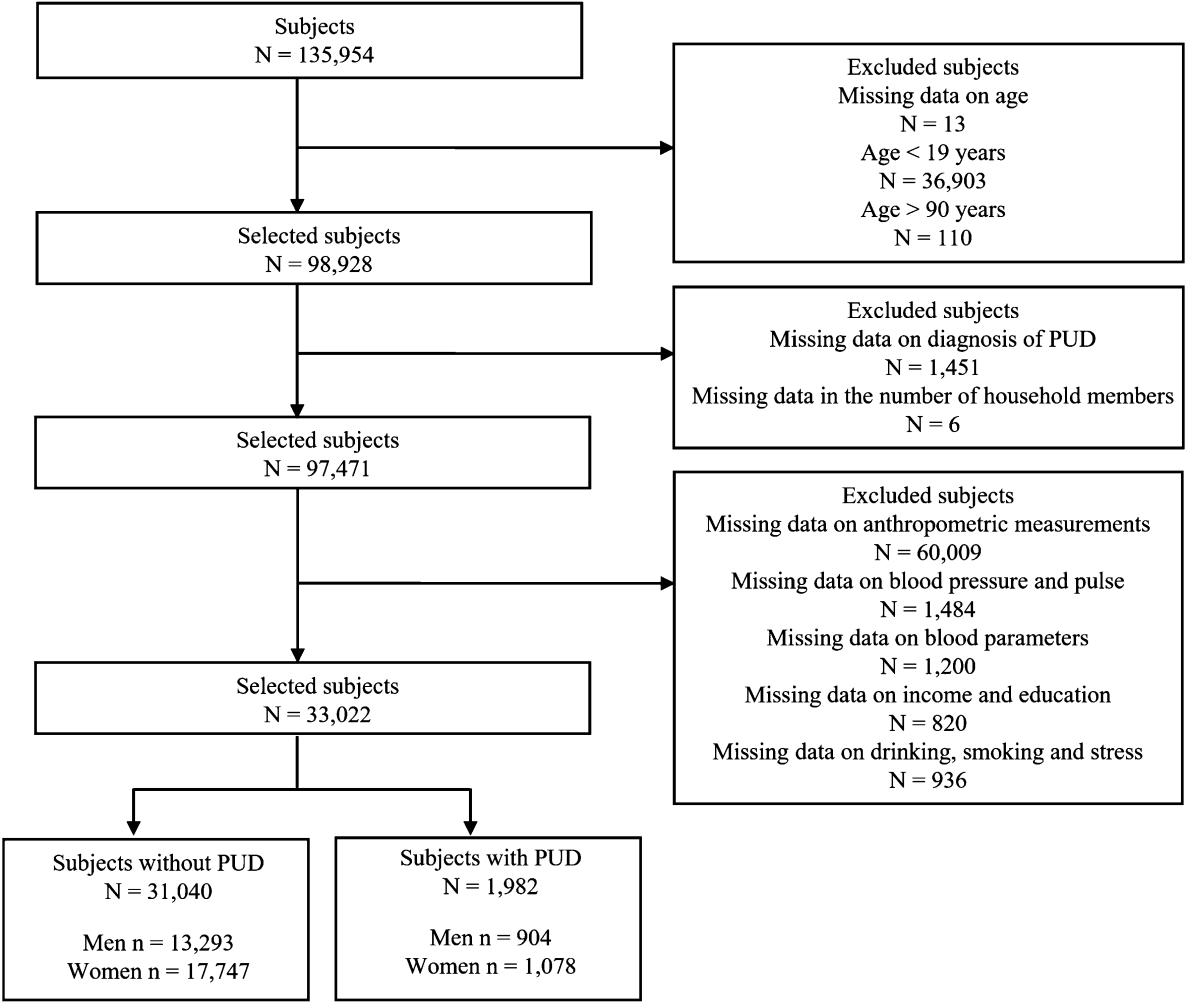
Sample selection flow. PUD: peptic ulcer disease.

The two component surveys of the KNHANES I-IV, health interviews and health examinations, included 135,954 subjects (men = 103,134, women = 32,820) enrolled from 16 representative cities in the Republic of Korea. Subjects were selected based on inclusion and exclusion criteria. We selected a total of 33,022 subjects: 31,040 subjects without PUD and 1,982 subjects with PUD. Details on the inclusion and exclusion steps and the number of subjects are shown in Fig. 1. All subjects in this survey signed informed consent forms. This study obtained ethics approval from the Institutional Review Board of the Korea Institute of Oriental Medicine for the analysis of the open source database KNHANES I-IV (IRB No. I-1909/007–003). The KNHANES was approved by the Korean Ministry of Health and Welfare and conducted in accordance with the Declaration of Helsinki^52^.

### Definition of PUD

Subjects with PUD were identified via the question “Do you have PUD diagnosed by a physician?” in a self-administered questionnaire. The PUD group consisted of subjects who checked “Yes”, and the non-PUD group consisted of subjects who checked “No” or “Not applicable” according to the KCDC guidelines. Specifically, the diagnostic definition of PUD included gastric duodenal ulcers and gastritis in the KNHANES I and gastric and duodenal ulcers in the KNHANES II-IV. Therefore, if subjects had at least one of the three ulcer types, they were included in the PUD group.

### Measurement and blood test

The variables in the two surveys, health interviews and health examinations, were used in this study to evaluate the association between PUD and the number of household members. We considered variables on socioeconomic status, medical condition and health behaviors in health interviews and variables on anthropometric measures, blood pressure, pulse rate and blood tests in health examinations.

Information on socioeconomic status (the number of household members, income, and education) and medical condition (gastric cancer, liver cancer, colorectal cancer, peptic ulcer diabetes, and hypertension) was collected via a self-administered questionnaire. Subjects with a disease such as gastric cancer, liver cancer, colorectal cancer, diabetes or hypertension were identified if they had been diagnosed with one of these diseases by a physician. Information on health behaviors (drinking, smoking, and stress) was collected using face-to-face interviews in the health interview questionnaire^52^. Alcohol drinking was categorized as subjects who had been drunk more than once during the last year or never drunk during the last year. Smoking was categorized as subjects who were smoking currently, quit smoking, or had never smoked.

Anthropometric measures (BMI, waist circumference), blood pressure and pulse rate, and blood tests (hemoglobin, cholesterol, etc.) were examined according to standardized protocols by trained medical personnel. Blood tests were performed using blood samples obtained after fasting for at least eight hours (Advia 1650, Siemens, New York, USA; Hitachi Automatic Analyzer 7600, Hitachi, Tokyo, Japan). The equipment used was calibrated periodically. BMI was determined by weight and height, and waist circumference was measured at the midpoint between the iliac crest and the lowest rib. Blood pressure was calculated as the average value of the second and third values of three measurements using a mercury sphygmomanometer (Baumanometer; Baum, New York, USA).

### Statistical analysis

All statistical analyses were performed using complex sample procedures in SPSS 23 for Windows (SPSS Inc., Chicago, IL, USA) to account for the complex sampling survey data. All statistical analyses were performed using weights to obtain estimates that were representative of Korean population to account for the complex sampling design. The weights with stratification were provided by the KNHANES. The significance level of α = 0.05 was used for all statistical tests.

Continuous variables are summarized as the means ± standard error (SE), and categorical variables are summarized as percentages (SE). General linear models were used for continuous variables, and Rao-Scott chi-squared tests were used for categorical variables to compare differences between the PUD and non-PUD groups. The same methods were used to compare differences across categories of the number of household members according to variable type.

Binary logistic regressions were used to investigate the association of PUD with the number of household members for each sex after the data were transformed by standardization. Multiple binary logistic regression models were constructed to analyze the association between PUD and the number of household members with different combinations of adjustment variables, taking into account several confounders that affected PUD in previous studies. Model 1 was adjusted for age and BMI as covariates. Model 2 was adjusted for age, BMI, income, location, and education, and model 3 was adjusted for age, BMI, income, location, education, and stress as covariates. Odds ratios with 95% confidence intervals and *p* values were indicated by sex.

## Data Availability

Data are available from the Korea National Health and Nutrition Examination Survey of the Korea Centers for Disease Control and Prevention (http://knhanes.cdc.go.kr/ and https://knhanes.cdc.go.kr/knhanes/sub03/sub03_02_02.do.
